# Military Importance of Natural Toxins and Their Analogs

**DOI:** 10.3390/molecules21050556

**Published:** 2016-04-28

**Authors:** Vladimír Pitschmann, Zdeněk Hon

**Affiliations:** 1 Faculty of Biomedical Engineering, Czech Technical University in Prague, Sítná sq. 3105, 272 01 Kladno, Czech Republic; pitschmann@oritest.cz; 2 Oritest spol. s r.o., Staropramenná 17, 156 00 Prague, Czech Republic

**Keywords:** toxins, toxin weapons, biological weapons, chemical weapons

## Abstract

Toxin weapon research, development, production and the ban on its uses is an integral part of international law, with particular attention paid to the protection against these weapons. In spite of this, hazards associated with toxins cannot be completely excluded. Some of these hazards are also pointed out in the present review. The article deals with the characteristics and properties of natural toxins and synthetic analogs potentially constituting the basis of toxin weapons. It briefly describes the history of military research and the use of toxins from distant history up to the present age. With respect to effective disarmament conventions, it mentions certain contemporary concepts of possible toxin applications for military purposes and the protection of public order (suppression of riots); it also briefly refers to the question of terrorism. In addition, it deals with certain traditional as well as modern technologies of the research, synthesis, and use of toxins, which can affect the continuing development of toxin weapons. These are, for example, cases of new toxins from natural sources, their chemical synthesis, production of synthetic analogs, the possibility of using methods of genetic engineering and modern biotechnologies or the possible applications of nanotechnology and certain pharmaceutical methods for the effective transfer of toxins into the organism. The authors evaluate the military importance of toxins based on their comparison with traditional chemical warfare agents. They appeal to the ethics of the scientific work as a principal condition for the prevention of toxin abuse in wars, military conflicts, as well as in non-military attacks.

## 1. Introduction

The term poison, in use since ancient times, means any substance (or mixture of substances) capable of inducing an adverse response in a biological system. The term toxin is rather more complicated. It came up only at the end of the 19th century, after the discovery of diphtheric toxin [1], and it has been traditionally employed for toxic protein substances, particularly those produced by microorganisms and certain animals (e.g., snakes); it was characterized by the presence of antigenic properties. However, the expansion of the knowledge of toxic substances obtained from natural sources, which also included many non-protein substances, brought about the need to consider a new definition of the term toxin [2]. In accordance with contemporary concepts, toxins are extremely poisonous products of the metabolism (independently of their nature) of living organisms; *i.e.*, bacteria (bacterial toxins), plants (phytotoxins), animals (zootoxins), and fungi (mycotoxins) [3]. These are individual chemical compounds of natural origin, which can be (and are) prepared by chemical or biotechnological procedures. The often used terms natural toxin and biotoxin reflect this classification. The term toxin is nevertheless also used, in an unsuitable manner, for exclusively synthetic chemical compounds, including extremely toxic chemical warfare agents (CWA).

One of the important characteristics of toxins is that they serve living organisms as a protection from predators or as a tool for hunting and killing prey. Their use in wildlife is part of the passive or active struggle for survival in certain species. As soon as people became acquainted with the special effects of natural poisons on living organisms (without understanding their principle), they started using them for their own purposes. They employed them to treat different diseases, to increase resistance to mental and physical fatigue, to reduce feelings of hunger, for magic and religious rituals, for hunting animals, for protection from troublesome insects and from dangerous animals, *etc.* Since time immemorial, people have also used natural toxins to commit suicide, murder, and also to wage war. They invented different methods for the propagation of natural poisons for military purposes, but only the development of munitions and carriers made it possible to efficiently use them in the form of toxic aerosols.

The present article is focused on selected problems associated with the possible military use of toxins and appropriate protection against them. It is based on the fact that the research, development, production and use of toxins for military purposes are strictly prohibited by international conventions and legislation in a vast majority of countries. The authors, however, believe that even under these beneficial conditions, it is desirable and purposeful to acquaint the scientific community and professionals with basic facts concerning the history of the military development of weapons based on toxins (toxin weapons) as well as with the state of art and possible risks resulting from the dynamic development of science and modern technologies, particularly biotechnologies. The authors are convinced that the consequent implementation of international conventions and qualified, sufficiently effective defense and protection against risks of the possible use of toxins in wars, military conflicts, or non-military attacks at any time in the future cannot be provided without the knowledge of history and a deep study of contemporary scientific and technical trends. In other words, the problems will not be solved by avoiding a relevant discussion. Deep knowledge free of myths and speculation, together with the ethics of the scientific work, are a basis of preventing any abuse of toxins and also enhance the trust in the sufficient efficacy of provisions adopted for the protection against any possible use of toxins.

## 2. Historical Notes

### 2.1. Poisoning of Water and Foods

Indigeneous tribes living essentially on all continents developed a technique of fishing by poisoning water with the use of plant extracts exerting dazing effects. For example, in Asia, the juice of *Derris elliptica* roots, containing rotenone, was used. North-American Indians from the Californian area used extracts from plants of the genus *Croton* containing phorbol for fishing [4]. This substance was also present in relatively-complicated African piscicides (substances poisonous to fish), typically based on plants of the genus *Euphorbia* [5]. Certain tribes also poisoned the water-holes of wild animals. In ancient times, these techniques using diverse natural materials were also used in armed conflicts and wars. The most popular case of the intentional contamination of water sources in military operations comes from ancient Greece, from the period about 600 BC. Ancient authors Frontinus (*Strategemata*) and Pausanias (*Hellados Periegesis*) describe the poisoning of the river Pleistos, the source of drinking water for the city Kirrha, by military troops of the League of Delphi with extracts from the hellebore (*Helleborus* spp.), which contains a number of toxins with cardioactive and spastic effects (hellebrin, ranunculin). Florence chronicler Giovanni Villani (*Nuova Cronica*) reports the poisoning of a drinking water source with hemlock (*Conium maculatum*) in a war of Florence and allies against Verona in the 14th century. Similar events accompanied our history for centuries.

Food was also subjected to deliberate contamination with toxins for military purposes. The origin of this technique can be found in indigenous tribes, for protection from wild animals by using poisoned baits. Numerous cases of poisoning food have been described as a stratagem since antiquity. A case from the Middle Age (11th century) is known, where Scottish troops poisoned food in the encampment of the invading Norwegian army with the juice from *Atropa belladonna* containing tropane alkaloids [6].

### 2.2. Poisoned Weapons

**Figure 1:**
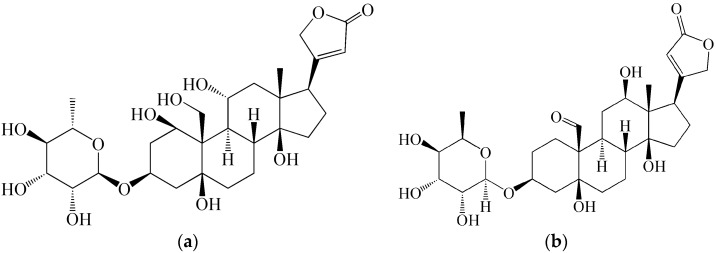
Chemical structures of (**a**) ouabain and (**b**) antiarin.

Observation of the nature and possible attacks of snakes or other actively poisonous animals led people to the discovery of arrow poisons as early as the Paleolithic era, used for soaking the tips of arrows and spears to enhance their efficacy [5,7]. This use was originally intended for hunting, but later (frequently also in modified compositions) they were also used in fighting. Poisoned arrows and spears do not cause mass destruction like modern toxin weapons, but they constitute complex systems containing a toxic substance and technical means for its transfer to the target. They can thus be considered as their first historical predecessors. The basic components of arrow poisons most frequently included plant toxins with different mechanisms of action and clinical pattern of effects. In spite of this diversity, the fact is of interest that arrow poisons based on alkaloids and glycosides were most frequently used. The alkaloids include for example aconitine, or strychnine, but particularly toxiferine I, tubocurarine and other neuromuscular blockers present in South-American arrow curare toxins. Glycosides with strong cardioactive effects (ouabain, convallatoxin, antiarin) can be found in arrow poisons known from Africa and Asia. An overview of certain non-protein plant toxins contained in arrow poisons is given in Table 1. As also documented by structural formulae of the ouabain (family Apocynaceae) and antiarin (family Moraceae) in Figure 1, arrow poisons were developed based on the same or very similar toxic principles on different continents. In certain areas, arrow poisons based on toxins produced by poisonous snakes, insects, or amphibians were used as well; arrow poisons of South-American Indians prepared from the skin secretions of frogs from the family Dendrobatidae are likely to be most famous. In addition, many of the arrow poisons contained irritating admixtures facilitating the absorption of toxins into the organism, and substances causing their fixation and preservation, which made their toxic and pharmacological effects last sometimes for several tens of years. In the period of modern science and technology development, arrow poisons were studied for their pharmacological and therapeutic characteristics, but neither has the idea of their military use vanished. In the countries of the so-called Third World, they are still used in military conflicts.

### 2.3. Toxin Ammunition

#### 2.3.1. Beginning of the Use of Toxin Ammunition

In historical military literature, it has been proposed that in the area of the Tigris and Euphrates rivers, “living grenades” were used in wars in the pre-historical period in the form of beehives and hornet nests [8]. Frontinus (*Strategemata*) reports that the Carthaginian military leader Hannibal recommended throwing vessels with poisonous snakes against the enemy´s boats in the second century BC. According to the Greek historian Herodian (*History of the Empire from the Death of Marcus*), at the end of the 2nd century, defenders of the Mesopotamian city Hatra threw clay vessels filled with scorpions and other poisonous animals against the Roman army. The same technique was also employed by warriors in the Middle Ages (for example the English king Richard I, also known as “Richard the Lionheart”) as well as in the Modern Age (for example during the Thirty Years’ War) [9,10].

Old-Indian strategist Kautilya (*Arthashastra*) (3rd to 4th centuries BC) gives instructions for the military use of toxic smokes obtained by burning poisonous plants, insects, snakes, and other animals. In the list of recommended substances, there are also the seeds of *Ricinus communis* or *Abrus precatorius* containing ricin or abrin, respectively. The burning mixtures were thrown either by hand or with the help of catapults. In the period of the Dong Dynasty (9th to 11th centuries), Chinese soldiers used toxic smoking balls of hemp fibres and dried toxic plants (for example *Aconitum* spp., *Croton* spp.), possibly enriched with snake venoms [11]. Similar toxic balls were also used by Mongolians [12]. With the introduction of gunpowder and artillery in Europe, grenades filled with the extracts of toxic plants (*Atropa belladonna*) or producing toxic smokes were encountered [13]. Several projects of ammunition filled with dried powders of the plants *Capsicum* spp., *Piper niger* or *Veratrum* spp., designed for the artillery or for dropping from balloons, were implemented in the course of the American Civil War [14].

#### 2.3.2. Toxin Ammunition in the Present Period of Modern Industrial Wars

World War I brought a dramatic development of CWA and their mass use in the battlefield. Chemical programmes of warring countries included not only synthetic CWA, but also natural toxins. As early as at the beginning of the war, British scientists carried out research into the military use of nicotine (*Nicotiana* spp.), veratrine (*Veratrum* spp.), and capsaicin (*Capsicum* spp.), and based on these alkaloids, they also proposed experimental ammunition [15,16]. The American Chemical Warfare Service proposed urushiol for filling artillery grenades; a toxin obtained from plants of the family Anacardiaceae that causes blisters on contact with the skin, similar to mustard gas or lewisite [17]. They also performed experiments into the possibility of using the toxic plant protein ricin as a possible replacement of phosgene and diphosgene. Two types of ammunition were proposed: shrapnels, where the toxin penetrates into the organism due to injuries with fragments, and grenades producing toxic aerosol. However, experiments demonstrated that efficient inhalation effects are difficult to achieve [18].

In the course of World War II, research on toxins was implemented within the framework of biological programmes. The USA tested botulinum toxin (*Clostridium botulinum*) filled into four-pound aircraft bombs, but the inhalation effects were poor; most experimental animals were poisoned due to injuries with contaminated fragments [9]. In spite of this, large amounts of botulinum toxin (X) were produced and stored until the end of the war. The research on ricin (W) continued, including a series of field tests with four-pound aircraft bombs and spraying devices filled with a ricin solution in water, with its suspension in tetrachloromethane or with raw ricin ground to fine powder. The tests demonstrated a low stability of the aerosol, low thermal stability of the toxin, and obviously also its insufficient purity. The industrial manufacture of ricin by pressing the ground seeds of *R. communis* under pilot plant conditions in the USA did not exceed 1.7 tons [18].

In the course of World War II, Great Britain together with Canada developed special aircraft bombs filled with numerous poisoned small arrows, which were scattered in the air and hit an area of 100 ares [19]. Canadian specialists preferred simple small arrows with poisoned tips. British specialists preferred small arrows in the shape of a sharp fountain pen filled with toxin. Botulinum toxin and other natural toxins were considered. Two methods of their dispersion were proposed. The first method concerned a bomb filled with about 36,000 small arrows, which hit soldiers horizontally at low elevation. The second type of bomb was in the form of a cassette filled with 30,000 small arrows scattered vertically at an elevation of about 900 m and hitting soldiers hidden in trenches. Experiments on animals demonstrated highly devastating effects of these weapons, but in spite of this, they were not included in the armament [19]. In the context of the British programme of toxin weapon development, there is information that a hand grenade filled with botulinum toxin was used during the assassination of Reinhard Heydrich, the Acting Reich-Protector of Bohemia and Moravia [20]. The assassination was carried out in May 1942 in Prague by Czechoslovak parachutists trained and armed in British special training camps. However, the use of botulinum toxin during the assassination was never confirmed.

The development of biological ammunition also continued after World War II. For example, in the USA, bomblets (for cassette bombs) were proposed that could comprise a suspension of ricin in tetrachloromethane [21] or certain toxins in the form of powder [22]. The possibility of putting toxins in warheads of tactical guided missiles or of applying them with the help of flying spraying devices was also considered. For an outline of certain types of biological ammunition with liquid and powdered filling developed in the USA in the 1950s to 1960s, see Table 2. Similar types of ammunition were also developed and tested in the Soviet Union (for example American bomb E120 corresponds to Soviet bomb Gshch-304) [23]. Certain developing countries were later also interested in toxin weapons. For example, Iraq, in around 1990, produced large amounts of botulinum toxin suspension, and aflatoxin solution, and they probably also considered the possibility of a military use of ricin. Iraqi specialists also used flying bombs, warheads of artillery rockets, and shells in tests.

#### 2.3.3. Weapons for Special Purposes

In the course of World War II, in the German concentration camps Sachsenhausen and Buchenwald, experiments with pistol projectiles poisoned with nicotine or aconitine were performed [25], which were to be used by special military troops. Japanese biological equipment (Unit Ei 1644 Nanjing, Unit 100 Changchun) tested snake toxin of the species *Naja atra*, insect poisons, toxins of certain species of sea fish, and ricin on humans [26]. The Military Medical Academy in Tokyo performed experiments with tetrodotoxin (fugutoxin) [27]. It is assumed that at least a proportion of these experiments were motivated by attempts to develop a toxin weapon for special purposes. The US Army Chemical Corps developed small gun arrows for special operations (“flechettes”) and gun projectiles with botulinum toxin or saxitoxin (TZ) in the 1950s to 1960s. Certain amounts of this ammunition were stored in the Pine Bluff Arsenal. A special laboratory of NKVD (KGB) in the Soviet Union also dealt with the development of similar products—for example, a weapon in the shape of an umbrella shooting miniature projectiles with ricin [28].

## 3. Contemporary Opinions Concerning Possibilities of the Military Use of Toxins

### 3.1. Toxins Most Important in Terms of Their Military Use

For military purposes, protein as well as non-protein toxins, occurring essentially at all levels of living organisms, can be used. As already mentioned, some of them found their use in the past during the development of experimental toxin weapons. Numerous toxins have been described, but only a small proportion of them can be effectively used for military purposes. In general, the main limitation results from a relatively tedious production process when it comes to industrial scale, in spite of the fact that some toxins can be produced in a simpler manner compared to classic CWA and that the availability of toxins is ever-increasing. The second key moment is their difficult application under battle conditions, which would make it possible to induce mass poisoning in the battlefield. For an outline of certain interesting military toxins and their comparison with the most important CWA, see Table 3. Some important differences between toxins and classic CWA are shown in Table 4.

#### 3.1.1. Plant Toxins

The vegetable kingdom offers a great variety of substances with considerable biological activities, which are typically produced as final products of the metabolism. Secondary metabolites with considerable toxic effects include terpenes, glycosides, alkaloids, amines, phenols, and other organic compounds. Plant toxins, as we have seen, serve as a basis of arrow poisons and they have long been used as parts of different tactical warfare mixtures. Some of them were recently subjected to military research. For example, the alkaloid physostigmine, isolated from the species *Physostigma venenosum,* served as a comparative standard in the development of synthetic inhibitors of acetylcholinesterase (AChE) or possibly in the development of protecting means against them. A less numerous but important group of metabolites in terms of their military use are toxic proteins. The top attention is focused on ricin or possibly abrin, modeccin (*Adenia digitata*), viscumin (*Viscum album*) or volkensin (*Adenia volkensii*), which have a similar structure, similar mechanism of toxic action (inhibition of protein synthesis), and almost identical clinical course of intoxication.

LD_50_ values (mouse, i.v.) for ricin are lower by a factor of five compared to VX and lower by a factor of more than 30 compared to sarin. In spite of the fact that the inhalation lethal dose of ricin for humans is at the level of sarin, the protection from ricin aerosol in the form of powder or solutions does not pose special difficulties. Pure ricin is readily soluble in water only, which is frozen at low temperatures. The suspension of ricin in tetrachloromethane (filling of experimental bombs) is unstable and affects the ballistic parameters of the ammunition. All forms of ricin are very sensitive to ultraviolet radiation, and thus its use in combat is problematic in periods of high solar activity. A certain handicap of ricin is also its slow action (toxic effect occurs after 8 to 72 h), which excludes operational-tactical use under the conditions of modern mobile combats. These disadvantages of ricin are not outweighed even by its easy preparation [32].

#### 3.1.2. Animal Toxins

**Figure 2:**
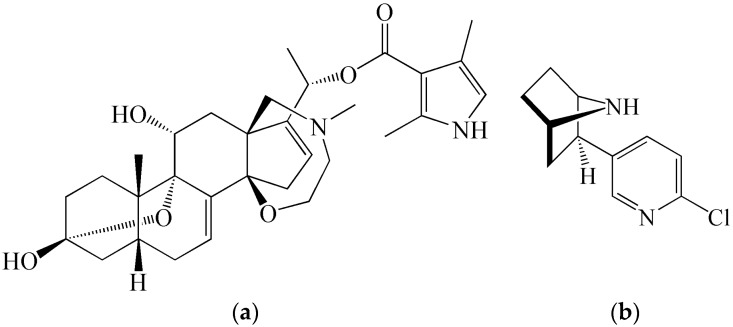
Chemical structures of (**a**) batrachotoxin and (**b**) epibatidine.

An important source of protein as well as non-protein toxins exerting a variety of toxic effects is also the animal kingdom. Certain poisonous animals have a special organ (gland) producing toxins (for example snakes, spiders, scorpions or a group of actively poisonous sea animals), some others do not have such an organ and their toxins are products of their metabolism or possibly parts of the biochemical structure of certain body parts. A proportion of animals acquire toxins from their environment (for example from their food) and the toxins are subsequently accumulated in their bodies. The military use of animal toxins is limited compared to plant toxins due to the fact that there are usually problems with their manufacture on a large scale [33]. However, this handicap is gradually overcome due to the development of new methods of chemical synthesis and biotechnology [34,35]. Animal toxins, which can be of interest for military use due to their high toxicity, include batrachotoxin, epibatidine (Figure 2), zetekitoxin AB and other alkaloids from the skin secretions of frogs from the families Dendrobatidae and Bufonidae, some active components of snake venoms such as taipoxin (*Oxyuranus scutallatus*), bungarotoxin (*Bungarus multicinctus*), and α-cobratoxin (*Naja siamensis*), spider toxins, for example α-latrotoxin isolated from the species *Latrodectus mactans,* and other toxins of terrestrial animals.

#### 3.1.3. Marine Toxins

An interesting group of toxins are those of marine origin produced by toxicogenic algae, cyanobacteria, and bacteria, which are common food of marine animals (certain species of fish, crustaceans, and molluscs), in which they are deposited (due to this, they are sometimes considered as animal toxins). These are non-protein substances, inducing alimentary poisoning in man and animals with different syndromes: paralytic shellfish poisoning (PSP), neurotoxic shellfish poisoning (NSP), amnesic shellfish poisoning (ASP), diarrheic shellfish poisoning (DSP), ciguatera fish poisoning (CFP) and azaspiracid shellfish poisoning (ASP) [36]. The highest risk is represented by neurotoxins with specific effects on the nervous system (saxitoxin, tetrodotoxin, palytoxin, maitotoxin, α-conotoxin, and others), which also exert a considerable military potential.

**Figure 3:**
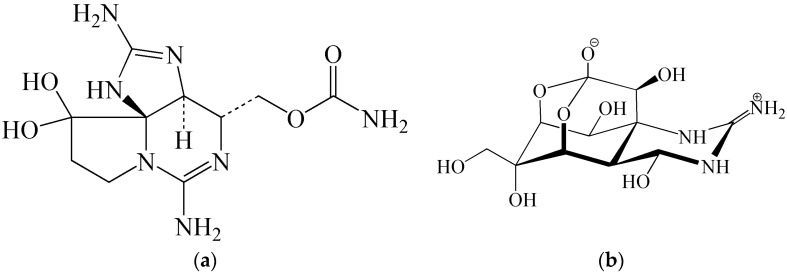
Chemical structures of (**a**) saxitoxin (STX) and (**b**) tetrodotoxin (TTX).

Marine toxins also have a high inhalation efficacy and thus their filling into ammunition producing toxic aerosols was considered. As early as the late 1980s it was found that the inhalation administration of saxitoxin (STX) to mice is about 10 times more efficient compared to intravenous administration. This is explained by the fact that in inhalation, the toxin effect is focused on a particular organ (lungs) and not on the organism as a whole, which is the case in intravenous administration. The inhalation lethal concentration (LCt_50_) of saxitoxin for man is about 5 mg·min/m^3^; *i.e.*, twice as low as for the substance VX and 15 times lower than for sarin. The saxitoxin aerosol is, however, very instable. It degrades at a rate of about 17% per minute. Saxitoxin (similar to low-molecular weight toxins tetrodotoxin, palytoxin, brevetoxin, anatoxin, T-2 toxin) is also able to overcome the dermal barrier, but it does not reach the efficacy of organophosphate CWA. The value of the percutaneous lethal dose of saxitoxin for man is unknown, but the analogue brevetoxin can be considered to act similarly. Experiments demonstrated that even a percutaneous dose of brevetoxin higher than its lethal parenteral dose by a factor of 20 does not cause any lethal poisoning [2]. Since the most interesting marine toxins, particularly saxitoxin, tetrodotoxin (Figure 3), or palytoxin are difficult to produce on an industrial scale, the possibilities of using high-capacity chemical (toxin) ammunition is limited [33]. Historical experience suggests that the most likely applications of toxins are in the field of manufacturing small-arms ammunition or in aimed sabotage acts and terrorist attacks focused on food contamination [37].

#### 3.1.4. Bacterial Toxins

Protein toxins produced by microorganisms induce a number of bacterial diseases (for example diphtheria, botulism, tetanus). Their biochemical structure is considerably diverse; they can be in the form of simple chains or substances comprising several units. Based on the chemical composition, thermostability, and method of release as a pathogen, bacterial toxins can be divided into two groups: exotoxin (toxic bacterial proteins) and endotoxins (toxic lipopolysaccharides) [33]. Exotoxins, which are considerably more toxic than endotoxins, are released by bacterial cells into the surrounding environment; endotoxins are, in contrast, bound to the cell wall of gram-negative bacteria. Certain exotoxins (for example diphtheric) are released from the bacterial cell in the form of non-active protoxin, which is subsequently activated by proteolytic splitting [30]. Depending on their mechanism of action, bacterial toxins can be divided into toxins damaging membranes (for example α-toxin), toxins inhibiting protein synthesis (for example shiga toxin), toxins activating second messenger pathways (for example cholera toxin), activators of immune response (for example staphylococcal enterotoxins), and proteolytic toxins (for example botulinum toxin, tetanus toxin) [38]. In experiments on animals, the lowest LD_50_ values are found in botulinum toxin and tetanus toxin, which have similar structures (belonging to the most complex known toxins), enzymatic activity, as well as effects on cells of the nervous system.

**Figure 4:**
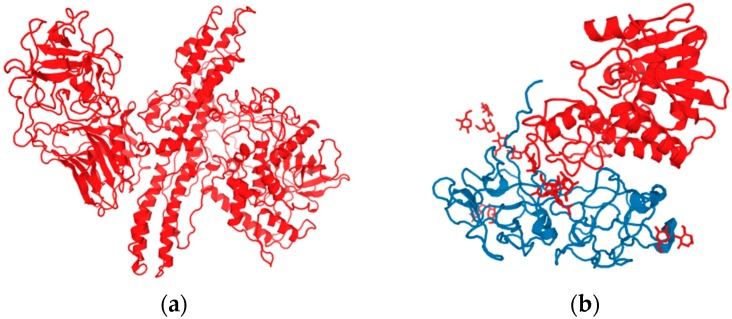
Structure of (**a**) botulinum toxin (A) [39] and (**b**) ricin [40].

The highest risk of abuse is particularly associated with toxic exotoxins, such as botulinum toxins, staphylococcal enterotoxins, diphtheric toxin, tetanus toxin, and shiga toxin. Some of them can be employed for poisoning water and food, or for producing fragment ammunition (poisoned weapons). Certain toxins can also act in the form of aerosols for inducing mass inhalation intoxications on larger areas, but their use is restricted. For example, botulinum toxin or its type A toxin, which is most interesting in terms of the military use (Figure 4 shows its structure compared to ricin), is more effective by a factor of about 100 than the substance VX (LCt_50_ of botulinum toxin through inhalation is 0.02–0.1 mg·min/m^3^), however, it is less suitable for combat use. The basic problem results from the aerosol’s stability. The most effective fractions (smaller than 10 µm) are dispersed in the upper layers of the atmosphere. The fractions reaching the Earth’s surface are then too large and do not have the efficacy required. Under comparable conditions, the area hit by aerosol of the substance VX is at least three times larger compared to the area hit by botulinum toxin. In general, in a long-distance transfer of aerosol through the atmosphere, an active portion of the toxin is degraded by the action of the surrounding environment. A disadvantage of botulinum toxin is also its poor capability to penetrate through the skin, and the possibly long latent period of the effect [32].

#### 3.1.5. Mycotoxins

Toxins are also produced by microscopic and macroscopic fungi. Particularly mycotoxins, low-molecular weight secondary metabolites of microscopic fungi that are responsible for various mass food poisonings (mycotoxicoses) can also be of importance from the viewpoint of military applications. The advantage of mycotoxins is the ease of their production on a large scale (by cultivation techniques or by synthetic methods) and their generally high stability (they are resistant to boiling). However, the existing military experience with several mycotoxins cannot be directly applied to the whole group of mycotoxins, since they have different chemical structures, biological characteristics, and effects (cytotoxic, neurotoxic, immunosuppressive, *etc.*) [41].

**Figure 5:**
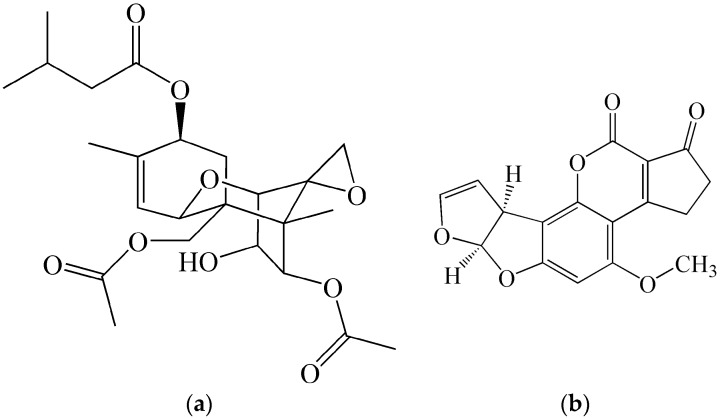
Chemical structures of (**a**) toxin T-2 and (**b**) aflatoxin B1.

Enhanced attention of military specialists was given to ergot alkaloids (*Claviceps purpurea*), aflatoxins (*Aspergillus* spp.), and over the last decades, to trichothecenes (*Fusarium* spp.), particularly a group including T-2 toxins (Figure 5 shows the structure of the T-2 toxin and its comparison with the structure of aflatoxin B1), HT-2 or diacetoxyscirpenol. Experiments on animals demonstrated that in the inhalation of finely dispersed T-2 toxin aerosol, lethal doses are lower by factors of 10 to 20 compared to parenteral administration. It is assumed that the LCt_50_ value for humans is around 5800 mg·min/m^3^; *i.e.*, higher by two orders of magnitude compared to sarin. After the application onto the skin, a general toxic effect of T-2 toxin is manifested, which is reminiscent of the blistering effect exerted by mustard gas [42]. The percutaneous lethal dose of T-2 toxin (LD_50_ for rat is 12.5 mg/kg) is at least 20 times higher than for the substance VX, and its action is much slower [32]. In addition, the toxin must be in the form of a solution, so the dilution factor has to be taken into account for achieving the toxic effect. It is known that in the early 1980s, the Soviet Union was accused of using weapons based on trichothecenes in the form of so called “yellow rains” in the wars in Afghanistan and South Asia [43]. The use of tricothocenes has not been confirmed. According to contemporary opinion, trichothecenes cannot be considered an ideal component of toxic weapons that could replace the most effective classic CWA. This, however, does not exclude their possible military use.

### 3.2. Toxins and Disarmament Conventions

Fifty years ago, toxins were still considered much more effective substances for attacking troops on a large scale than classic CWA. However, military research, particularly the results of tests with different types of ammunition used under field conditions, showed that they did not meet these expectations. Further development of toxins did not occur, since they were overtaken particularly by the development of nerve CWA. Historical experience with military research, development, and use toxins was also reflected by international disarmament documents: the Geneva Protocol of 1925, the Biological and Toxin Weapons Convention (BTWC) of 1972 and Chemical Weapons Convention (CWC) of 1993. These conventions (particularly the BTWC and CWC) belong to the historically most important disarmament actions, which considerably contributed to a reduction in the probability of using chemical, biological, and toxin weapons. They also established appropriate conditions for the enhancement of the protection against these weapons in moderation or in certain cases, even elimination of their destructive effects. Specific problems are nevertheless still persisting.

For example, the main drawback of BTWC is the persisting absence of control mechanisms. The convention also applies to toxins, but does not comprise their definition. Only at the second conference evaluating the BTWC in 1986 was it demonstrated that it concerns toxins (protein as well as non-protein ones) of microbiological, animal, or plant origin, and their synthetically-produced analogs. For purposes of the CWC, the term “chemical weapon” (*inter alia*) refers to weapons containing any toxic chemicals, which can cause death, temporary incapacitation, or permanent harm to humans or animals due to its chemical action on life processes. It includes toxic chemicals independent of their origin or method of production, *i.e.*, toxins as well. For purposes of the CWC implementation, the Schedules of Chemicals (Schedule 1) contains ricin and saxitoxin, *i.e.*, representatives of both basic types of toxins: protein and non-protein. However, the CWC is aimed at the use rather than at the chemicals (General Purpose Criterion), and thus it also covers substances which have not yet been discovered and synthesized and can emerge in the future with the aim of using them as chemical weapons. On the other hand, even the most toxic toxins can be manufactured in defined amounts and under international control (including toxins specified in Schedules) for purposes not prohibited by the convention, *i.e.*, for industrial, agricultural, research, medical, pharmaceutical (or other peaceful purposes), protection purposes, military purposes not connected with the use of chemical weapons and law enforcement including domestic riot control purposes.

### 3.3. Toxins and Terrorism

The scientific and technological progress and the availability of information about technologies of product synthesis suggest that there may be an enhanced likelihood of the non-military use of toxins and an increased awareness of their psychological effects. This can be referred to as the problem of chemical (biological) terrorism, which is part of a wider set of concepts called superterrorism or ultraterrorism. As far as toxins are concerned, this concerns their use (including attacks against facilities in which toxins occur) by individuals, non-state groups, or state-supported subjects against a particular social group to induce fear or terror. In the past, there were a number of planned or implemented attacks of such kind. For example, in 1980, the West-German terrorist group Red Army Faction was suspected to have planned using botulinum toxin, which was prepared in a private laboratory in Paris [44]. This kind of toxin was also used for experiments by the Japanese faction Aum Shinrikyo, known particularly for their sarin attack in the Tokyo metro [45]. The American nationalist anti-governmental movement Minnesota Patriots Council planned the use of ricin in dimethyl sulfoxide solution (with admixture of extract from the plant *Aloe vera*) or in the form of dry aerosol in 1991 [46]. In the course of the Chechnya war (2001), Russian authorities revealed a plan of local rebels who reportedly intended to use ricin in the form of mixed ammunition. It seems that ricin, when it comes to terrorism, has been recently considered as one of most frequently cited toxic substances [47].

The use of toxins for terrorist purposes is less probable than the use of industrial harmful substances or classic CWA, but the probability is higher than for military use. There are questions about their actual effects. Ricin as well as other known toxins are relatively easily accessible to any qualified specialist, but their use in terrorist attacks with the aim of inducing mass intoxications through inhalation is difficult. This reduces the probability of inhalation attacks. Terrorist attacks focused on drinking water and food sources, and particularly on the destruction of a particular person or group are more realistic. At the time of modern information, technologies and propagation of the fear of a global threat by weapons of mass destruction, toxins can serve as an efficient psychological weapon, even if they are not actually used.

### 3.4. Non-Lethal Weapons

There are a number of definitions of non-lethal chemical weapons. For example, in accordance with the NATO definition, these are “Weapons which are explicitly designed and developed to incapacitate or repel personnel, with a low probability of fatality or permanent injury, or to disable equipment with minimum undesired damage to or impact on the environment” [48]. Non-lethal weapons are currently a common supplement to conventional weapon systems, at least in most developed armed forces. One of the many non-lethal technologies used is based on toxic principles; in this case, the terms “non-lethal chemical weapons” is most frequently used, but there are also the terms “non-lethal biological weapons” or “non-lethal toxin weapons”.

#### 3.4.1. Toxins as Incapacitating Agents

**Figure 6:**
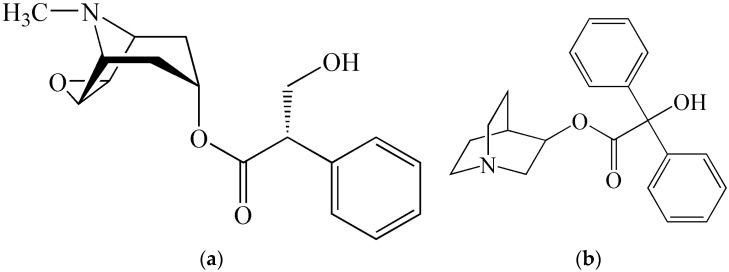
Chemical structures of (**a**) scopolamine and (**b**) its synthetic analog (non-toxin), agent BZ.

The active components of non-lethal chemical weapons are traditionally incapacitating agents with very diverse biological effects on the human or animal organism. They can cause physical incapacitation (different irritating agents, tremorgens, emetics, narcotic analgesics, dissociation anaesthetics, or neuroleptics), but also psychological incapacitation (sympatomimetics or anticholinergics). Many of these incapacitating agents were studied in the past as potential CWA (components of chemical weapons), and some of them were even introduced into the standard military armament (for example irritating agents CN, CS, CR, psychoactive agent BZ—all of them being synthetic compounds, non-toxins). In association with these agents, one can speak about non-lethal chemical weapons, but many of them belong to natural toxins or have chemical structures which are analogs of naturally-occurring substances. Natural alkaloid capsaicin or its synthetic analogs, e.g., vanillylamide of pelargonic acid (PAVA) or morpholide of pelargonic acid, can serve as examples of this relationship. Hallucinogen LSD-25, intensively studied in military laboratories fifty years ago, is a synthetic analog (diethylamide) of lysergic acid, which belongs to the ergot alkaloids. The structure and toxic (anticholinergic) effect of the synthetic psychoactive agent BZ are similar to those of tropane alkaloids, for example scopolamine (Figure 6).

Protein toxins, particularly staphylococcal enterotoxin B (SEB), an activator of immune response, were studied in the past as incapacitating agents (components of non-lethal biological weapons). This toxin induces strong vomiting, and so it can be compared with the strongest modern emetics already at effective doses (ED_50_) of 0.1 to 1 µg/kg (depending on the route of administration). In certain experiments, staphylococcal enterotoxin serves as a standard for the comparison of synthetic emetics developed as possible candidates for incapacitating agents.

The military potential of toxins is not necessarily associated with their lethal effects. Many toxins have unique biological effects, which can affect cognitive functions or intellectual capacities in humans. Domoic acid, a relatively simple compound close in structure to glutamic acid occurring in molluscs, can serve as an example of this. It is only moderately toxic (LD_50_ in mouse i.v. is higher than 10,000 µg/kg) [49], but due to the fact that it is bound to NMDA-glutamate neuronal receptors, it induces a number of neurological problems and causes permanent damage to short-term memory. This is not a necessary requirement for non-lethal chemical (biological) weapons, but the possible military use of domoic acid and other toxins exerting similar effects is nonetheless not excluded.

#### 3.4.2. Toxins as Riot Control Agents

The convention on CWC categories does not define “non-lethal chemical weapons” or “incapacitating agents”, in contrast to other relevant organizations [48]. However, it introduces a category of “riot control agents” (RCA), which means any chemical agents (not included in the Schedules) which are able to rapidly induce irritation of human sensory organs or to cause enormous physical effects which vanish over the short term after the end of exposure. Incapacitating agents, in contrast to RCA, can also cause degrading physical effects, which persist for a longer period after exposure. Their effects are thus more serious. The CWC convention explicitly prohibits the use of RCA as “a method of warfare”, without defining this term. This provision was considered as a prevention of the military use of these agents, as was the case, for example, in the war in Vietnam. In spite of this, there is a risk of their military use. Similar to World War I, the use of these agents can be a precedent, which can lead to a subsequent (also retaliatory) use of lethal agents for the escalation of chemical war. 

**Figure 7:**
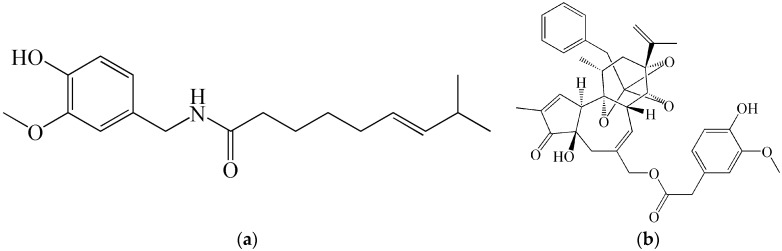
Chemical structures of (**a**) capsaicin and (**b**) resiniferatoxin.

In practice, these are (in addition to malodorants) the already-mentioned irritating agents. However, experience shows that there is a risk that this category can factually include not only irritating agents but also mentally and physically incapacitating agents with diverse characteristics and effects. For example, during the Moscow anti-terrorist intervention in 2002, synthetic opioids (non-toxins) derived from fentanyl (carfentanil, remifentanil) were used [50], which have incomparably more efficient calmative effects than the natural opiate morphine. These agents can obviously be not only the synthetic compounds mentioned, but also toxins. In police practice as well as for personal protection, the alkaloid capsaicin and its synthetic derivatives are used. The possibility exists that new toxins will be discovered or their analogs will be synthesized, exerting even more efficient incapacitating effects (irritating, psychoactive, calmative) which are not yet known. For example, resiniferatoxin, structurally more complicated than capsaicin (Figure 7), isolated from a species of *Euphorbia resinifera* or *E. poissonii* (total synthesis proposed in 1997) [51] effectively irritated the human skin even at extremely low doses (ED_50_) of 2.4 × 10^−7^–2.4 × 10^−9^ mg/cm^2^. This can have an important impact on their military use. The protection from aerosols of toxins and their analogs important in terms of their military use typically does not pose serious problems for appropriately-trained and technically-equipped military units. Isolation of the body surface completely eliminates effects of toxic aerosols and the currently used protective masks are designed in such a way that the concentration of aerosol penetrating into the space under the mask is lower by a factor of 100,000 to one million compared to the aerosol concentration in the external environment. A different situation can be encountered in using modern super-effective irritating toxins (possibly also resiniferatoxin). With certain new methods, under field conditions, it is possible to maintain concentrations of solid aerosols at a level of 1–10 mg/m^3^ for sufficiently long periods of time. In this case, super-effective toxins can cause lacrimation, cough, respiratory problems, and other undesirable effects, thus forcing the exposed individual to remove the mask and expose themselves to life-endangering concentrations [32]. These considerations could even result in a revision of requirements for the protective coefficient of gas mask. 

The development of incapacitating agents (including RCA) is in many respects analogous to the development of medical products with high therapeutic effects. There is a problem, also demonstrated by the experience from Moscow, that the use of agents incapacitating wide groups of persons having different degrees of the susceptibility (age, sex, health condition) without endangering their health is technically enormously difficult, if not impossible. Based on this experience, many specialists consider that there is a low probability of finding substances with sufficient safety coefficients [52]. This also means that the term "non-lethal" may be unsuitable for incapacitating agents [53].

#### 3.4.3. Bioregulators

Together with toxins, bioregulators are studied, which are naturally occurring substances, usually peptides, participating in physiological and neurological processes in the body. They affect for example blood pressure, myocardium activity, breathing, muscle contractions, body temperature, sleep, mood, emotions, immune reactions, *etc.* They are characterized by being active already in extremely low doses, their effects being very rapid. Bioregulators formally do not belong to the group of toxins, but they share a similar potential for military use [54]. Certain bioregulators can have similar structures and mechanisms of toxic actions as toxins (for example the bioregulator endothelin, which increases blood pressure, is similar to cardioactive safratoxins isolated from poison glands of snakes from the genus *Atractapis*). Drawbacks of bioregulators as potential new-generation chemical or biological weapons (particularly non-lethal chemical weapons or RCA) is their low resistance to ferments present in the human organism, for example, in the skin. The advantage of this fact can be taken in the protection against bioregulators and moderation of their toxic effects. Weapons based on bioregulators are sometimes referred to as hormonal weapons, and together with toxins, as biochemical weapons.

## 4. Certain Risks of the Further Development of Toxin Weapons

### 4.1. Research of Toxins

#### 4.1.1. Discoveries of New Toxins

Research on natural sources brings ever new discoveries of sometimes very effective toxins. In this respect, interesting knowledge is encountered in the field of marine pharmacology and marine toxicology. Hundreds of substances are found in the seas every year, which open new views of life processes in the still scarcely studied sea environment and offer applications in medicine and other fields beneficial to the human being [55]. Some of these substances of marine origin can also be of interest to military specialists. A number of discoveries of toxins of terrestrial plants or animals, which have similar chemical structures to marine toxins, were recently reported. Zetekitoxin AB, a structural analog of saxitoxin, can be mentioned by way of example, which was isolated from the Panama frog *Atelopus zeteki* [56].

#### 4.1.2. Preparation of New Low-Molecular Weight Substances and Peptides

The research of toxins is usually a part of the development of new medicinal products in biomedical and pharmaceutical laboratories. New medicinal products are traditionally discovered by the verification of biologically-active substances (including toxins or bioregulators) produced by living organisms. Once discovered, a substance can be modified chemically in different ways with a realistic hope that its biological activity will be enhanced. New pharmaceuticals are also currently prepared by methods of combinatory chemistry, which makes it possible to systematically combine different structural blocks and thus to obtain great sets (libraries) of structurally closely related low-molecular compounds and peptides. These libraries can be then used by ”high-throughput screening” for the identification of specific compounds, depending on the effect required [57,58]. There is always a possibility that among millions of newly prepared substances, new synthetic analogs of toxins with considerable military potential will occur. However, the toxicological data obtained are usually based on *in vitro* screening (in contrast to *in vivo* screening used in the past during the development of chemical and biological weapons), and so, they need not reflect the actual toxicity.

#### 4.1.3. Hybrid Toxins

An interesting trend in the development of protein engineering is represented by hybrid or modified protein toxins produced by a combination of binding and catalytic domains of two different toxins. The result of this is a toxin with programmed characteristics suitable for use in different fields. In agriculture, hybrid insecticide toxins are promising; for example, based on delta-endotoxin produced by the bacterium *Bacillus thuringiensis* [59]. In oncological therapy, immunotoxins may be beneficial, containing two domains, one of them having the nature of an antigen and the second of a toxin fermentative subunit. As the fermentative component, fermentation domains of toxins blocking syntheses of proteins were proposed, for example that of ricin [60]. In terms of military use, a hybrid toxin may be of interest which is obtained by a combination of botulinum toxin and staphylococcal enterotoxin, which is not only extremely toxic but also very stable in the outdoor environment [61].

#### 4.1.4. Problem of Maximum Toxicity

In certain groups of natural substances (including toxins interesting for military use) as well as synthetic compounds (including CWA), a certain increase in the biological activity was observed with increasing molecular mass. By modification of the chemical structure (introducing substituents), a certain increase in the toxicity can be actually achieved, which was also implemented in the past within the framework of the CWA development. However, this modification has its limits. In the case of a substantial increase in the toxicity (at least by an order of magnitude), structural changes in a certain type of the compound are insufficient and it is necessary to use a compound having a much higher molecular mass and a much more complicated structure. Presently, the biologically most active substance is botulinum toxin, the molecular mass of which (150 kDa) approaches the upper limit of possible molecular masses of proteins. Based on a theoretical analysis, it is possible to suggest that molecular masses of toxins with LD_50_ lower by more than an order of magnitude compared to botulinum toxin should reach values of about 1500 kDa (only few proteins with this molecular mass are known). If the toxins should be more toxic than botulinum toxin by two orders of magnitude, then they should have their molecular mass as large as 15,000 kDa, but such proteins have not yet been described. This means that the botulinum toxin toxicity is limiting not only for bacterial toxins but for natural toxins in general. The modification of any toxin will probably result only in a change in the spectrum of its effects [32,62]. On the other hand, the toxicity is an important criterion of the toxin suitability for military use, but this is not the only criterion and frequently is not even a decisive criterion.

### 4.2. Manufacture of Toxins

#### 4.2.1. Obtaining Toxins from Natural Sources

The basic method of toxin preparation is production directly from natural sources. Some procedures, such as the extraction of toxins from natural materials, fermentation, *etc.* are known and have been successfully used for thousands of years (for example, manufacture of arrow poisons); some others, for example biotechnological procedures, were relatively recently proposed and were supplemented by modern methods of isolation and purification. A proportion of toxins can also be obtained from natural sources in amounts sufficient for military use, as is the case for example with ricin, botulinum toxin, SEB, or capsaicin. The industrial production of certain toxins in this way can be, however, for economic, ecological and other reasons (e.g., attainability of natural sources) non-effective, if not impossible.

#### 4.2.2. Synthesis of Low-Molecular Weight Toxins

Many low-molecular weight toxins became attractive for military purposes only after it was possible to determine their chemical structure and develop a procedure of the total chemical synthesis. This knowledge reduces the dependence on natural sources (at least theoretically) and makes possible the production of toxins even on an industrial scale. As an example, it is possible to mention saxitoxin (STX) or a group of chemically related compounds referred to as saxitoxins (57 analogs were already known in 2010) [63]. Saxitoxin was originally prepared by the extraction from tissues of certain marine animals, but the yield was very low (only several grams from tons of biological material). This technology made possible the use of saxitoxin only in the form of poisoned projectiles or small arrows. Only the discovery of marine microorganisms as primary sources of saxitoxin made it possible to propose a modern biotechnological method of its production on a larger scale. In addition, based on knowledge of the saxitoxin chemical structure, procedures of chemical synthesis were proposed that were suitable for industrial applications; the first total synthesis was already proposed in 1977 [64]. Based on this progress, concepts of the military use of saxitoxin in the form of ammunition producing toxic aerosol became much more realistic. In addition, in laboratory and subsequently also in manufacturing plants, derivatives of saxitoxin can also be synthesized, which do not occur in nature and can exert certain characteristics interesting for military use. However, chemical synthesis is not always advantageous. For example, T-2 toxin was first isolated in the late 1960s, and based on knowledge of its chemical structure, a method of its synthesis was proposed, but the yield was poor. Its preparation by microbiological methods based on the cultivation of toxicogenic strains of fungi is more advantageous.

#### 4.2.3. Microtechnology

Considerable progress in the production technology of pure chemical and pharmaceutical compounds was achieved by using methods of chemical microtechnology [65]. The use of microprocessors and small-dimension flow reactors is prevalent in research and development laboratories, but they are associated with certain risks as to the implementation of conventions CWC and BTWC. This technology is also suitable for the manufacture of high-molecular weight toxins and their synthetic analogs, which can be performed in large amounts and in less easily identifiable equipment.

#### 4.2.4. Gene Engineering

In the late 1970s, there was a rapid development of gene technology, which now makes it possible to produce certain natural substances more easily than any time before. The fact that by methods of gene and metabolic engineering, used for example in the pharmaceutical or food industries, it is also possible to produce toxins more effectively, resulting in a new increase in the danger of their use as chemical or biological weapons. By genetic manipulation, specific genes important for toxin weapons can be put to a different place within the framework of the genome of a certain cell to achieve a higher gene expression. It should be taken into account that these genes can also be introduced into a cell of another (alternative) organism to provide toxin production or to establish conditions for its mass biotechnological production. In the far future, the biosynthesis of toxins interesting for military purposes can also occur in “artificial” (yet unknown) organisms produced by synthetic biology.

### 4.3. Applications of Toxins

#### 4.3.1. Microencapsulation

Microencapsulation is a technique of pharmaceutical administration in the form of microdroplets with a biologically degradable polymeric shell. The purpose of microencapsulation is the protection of the active substance from its degradation, the possibility of the control of its release into the organism, and simpler handling [66]. This technique was already proposed in the past for classic CWA and can also be advantageously used for toxins. The advantage of encapsulated toxins can be an enhanced stability of toxin aerosols in the atmosphere and an increase in their inhalation as well as percutaneous efficacy, which are the main problems accompanying the use of toxins by traditional methods.

#### 4.3.2. Nanotechnology and Aerosol Application

The development of nanotechnology involved fears that it can also be used for the development of chemical or biological weapons including toxins [67,68,69]. This fear was based on the assumption that nanoparticles exert high chemical and catalytic activity, which has not been observed in larger particles of the same substance, and achieve higher concentrations in the contaminated environment and easier penetration into the invaded human or animal organism. The assumption that nanotechnology will dramatically increase the efficacy of contemporary forms of CWA and toxins (or of their synthetic analogs) important for military use has not been confirmed, but relevant knowledge is still missing in this field. On the other hand, nanotechnology can considerably affect the efficacy of the transfer of biologically active substances to target objects. For example, the pharmaceutical industry develops nanoparticles as carriers of pharmaceuticals with inhalation effects, which are transported in the form of aerosol into the lungs and from there into the vascular bed. Nanoparticles can also be useful in a controlled release of medicinal products, in their penetration through the membrane barrier and in specific effects on cells or organs.

#### 4.3.3. Mixtures

Military applications of toxins in the form of tactical mixtures offer other possibilities of the military application of toxins. This technique was commonly used in the past for the adjustment of physical and chemical properties of CWA. Suitable admixtures increased the CWA stability, improved ballistic characteristics of the ammunition and provided more effective transfer of the agents to the warfare condition. Certain components added (as for example dimethyl sulfoxide) improved the absorption of CWA by the skin and thus increased their percutaneous effects. In military laboratories, CWA mixtures were also proposed which exerted unusual toxic effects and increased requirements for the chemical analysis, use of protective means, decontamination and therapy. Certain proposals of mixtures also included classic CWA in combination with toxins, for example mustard gas with aflatoxins. Enhanced effects can also be achieved by dissolving ricin in liquid CWA [32]. A concept of the application of mixtures of different toxins, which can present synergistic or more diverse effects, is also not unrealistic.

## 5. Conclusions

Today, perhaps more than any time before, it is of importance to study historical texts, military handbooks, and notes on the military use of natural toxic substances. Even the oldest information on sources of toxins, methods of their application, and destructive effects could inspire contemporary as well as future scientists dealing with the research of toxin weapons. Toxins important for military purposes (sometimes referred to as “toxin warfare agents” by analogy with “chemical warfare agents”) belong to the so called “mid-spectrum agents”. They are found in chemical and biological weapons, which have been subjected to several international disarmament conventions and thus, their military use to a considerable extent is not currently taken into account. In spite of the fact that in the past the development of toxin weapons was not successful compared to classic chemical weapons, and that it is currently forbidden by disarmament conventions, the research and use of toxins (as toxic chemicals) for activities which are not forbidden is further in progress. There is a problem that certain new trends in the development of chemical, biochemical, and biological technologies can undermine the intention of these conventions, because they may establish conditions for upgrading toxin weapons to a level which possibly enables their effective mass use. The development of toxin weapons (and within a wider concept, bioregulators) can be more easily concealed compared to the development of classic chemical weapons or biological weapons based on pathogens causing infectious diseases. It seems that classic chemical weapons have reached their zenith and possible risks of their further development can be at least anticipated. However, potential possibilities of the development of weapons based on toxins and their synthetic analogs pose quite new problems in the field of the protection and healthcare, including the detection, means of chemical protection, decontamination, first aid, diagnosis, and qualified therapy. A certain period of time is necessary for the development of any weapon, which also holds for toxin weapons. If we consider the development of toxins for military use to be similar to the development of any other biologically active product (including medicinal products), then it would take a period of 5 to 10 years. However, it can be expected to be carried out under conditions of concealment. This fact gives rise to fears that the world could implement appropriate countermeasures by developing effective methods and means of protection or legislative tools only after experiencing the consequences of their application.

## Figures and Tables

**Table 1:** Outline of certain non-protein plant toxins contained in known arrow poisons.

Toxin	Structure	Source (example)	Region of Use	LD_50_, µg/kg
Aconitine	Alkaloid	*Aconitum* spp.	Europe, Asia	100 (mouse, i.v.)
Antiarin	Glycoside	*Antiaris toxicaria*	Asia	900 (cat, i.v.)
Cicutoxine	Alcohol	*Cicuta* spp.	North America	~9000 (mouse, i.p.)
Convallatoxin	Glycoside	*Parquetina nigrescens*	Africa	200 (cat, i.p.)
Ouabain	Glycoside	*Strophanthus* spp.	Africa	110 (cat, i.v.)
Strychnine	Alkaloid	*Strychnos nux-vomica*	Asia	400 (mouse, i.v.)
Toxiferine I	Alkaloid	*Strychnos toxifera*	South America	20 (mouse, i.v.)
Tubocurarine	Alkaloid	*Chondrodendron* spp.	South America	100 (mouse, i.v.)

**Table 2:** Certain types of biological ammunition developed in the USA over the 1950s–1960s [2,24].

Type	Description
E44R2	Aerosol generator
A/B45Y-1	Aircraft sprayer of liquid receptures (for tactical aircrafts)
A/B45-4	Aircraft sprayer of dry receptures (for tactical aircrafts) (tested SEB)
M114	Submunition for M33
M33	Cluster bomb 500 pounds (108 pcs M114)
E61R4	Submunition for E133
E133	Cluster bomb 750 pounds (536 to 544 pcs E61R4)
M143	Submunition for M210
M210	Warhead of guided missiles
E120	Submunition for cluster bombs and warheads

**Table 3:** Outline of certain interesting toxins in terms of their military use and their comparison with most important chemical warfare agents (CWA) (LD_50_ values according to [2,24,29,30,31]). AChE: Acetylcholinesterase.

Toxins/CWA	Structure	Action Mechanism	LD_50_ (Mouse, i.v.) μg/kg	M_r_
Botulinum toxin	Protein	Inhibition of acetylcholine release	0.001	150,000
Tetanus toxin	Protein	Inhibition of GABA release	0.002	150,000
Maitotoxin	Polypeptide	Ca^2+^ agonist	0.1	3400
Palytoxin	Polyalcohol	K^+^ channel blocker	0.15	2700
Abrin	Protein	Protein synthesis blocker	0.7	65,000
Batrachotoxin	Steroid alkaloid	Na^+^ channel activator	2	539
Taipoxin	Protein	Inhibition of acetylcholine release	2	35,000
Ricin	Protein	Proteosynthesis blocker	3	62,000
Tetrodotoxin	*N*-heteroaromatic	Na^+^ channel blocker	8	319
Saxitoxin	*N*-heteroaromatic	Na^+^ channel blocker	10	299
Physostigmine	Alkaloid	AChE inhibition	450	275
T-2 toxin	Trichothecene	Inhibition of protein synthesis	1200	466
VX	Organophosphate	AChE inhibition	15	267
GD	Organophosphate	AChE inhibition	64	182
GB	Organophosphate	AChE inhibition	100	140

**Table 4:** Principal differences between toxins and classic CWA [2].

Characteristics	Toxins	Classic CWA
Origin	Natural	Synthesis
Manufacture	Complicated, low series	Heavy industry
State of matter	Solid	Liquid (lethal CWA)
Volatility	Non-volatile	Volatile
Toxicity	High	Lower
Percutaneous effect	Not characteristic	Yes
Taste and smell	No	Yes
Toxic effect	Diverse	Less diverse
Immunogenic properties	Partially yes	No
Aerosol by explosion or thermal	Problematic	Possible (fogs, droplets, smokes)
Individual protection	Anti-smoke filter	Mask and body protection means
Detection in real-time	Impossible, problematic	Possible
